# Evaluation of antibacterial properties and shear bond strength of orthodontic composites containing silver nanoparticles, titanium dioxide nanoparticles and fluoride: An in vitro study

**DOI:** 10.1590/2177-6709.27.5.e222067.oar

**Published:** 2022-11-07

**Authors:** Tivanani Venkata Durga MAHENDRA, Vizia MUDDADA, Suresh GORANTLA, Tarakesh KARRI, Vyshnavi MULAKALA, Ratnavati PRASAD, Sarath Kumar CHINTALA, Kotta MOUNICA

**Affiliations:** 1 Vishnu Dental College, Department of Orthodontics and Dentofacial Orthopedics, (Bhimavaram, Andhra Pradesh, India). dr.durgamahendra@gmail.com; 2 Sree Sai Dental College and Research Institute, Department of Orthodontics and Dentofacial Orthopedics (Srikakulam, Andhra Pradesh, India).; 3 Anil Neerukonda Institute of Dental Sciences, Department of Orthodontics and Dentofacial Orthopedics (Bheemunipatnam, Andhra Pradesh, India).; 4 Private practice (Visakhapatnam, Andhra Pradesh, India).

**Keywords:** Metal nanoparticles, Nanocomposite, Antibacterial activity, Shear strength

## Abstract

**Objective:**

The study aimed at determining the antibacterial properties of composites containing silver nanoparticles (Ag NPs) or titanium dioxide nanoparticles (TiO_2_ NPs), and a fluoride-releasing composite against *Streptococcus mutans* and *Lactobacillus acidophilus*, and to evaluate the effect on shear bond strength (SBS) of nanoparticles-modified composites.

**Materials and Methods:**

An orthodontic composite was modified by adding 1% w/w Ag NPs or 1% w/w TiO_2_ NPs. Composite discs were prepared to evaluate the antibacterial properties of these modified composites against *Streptococcus mutans* and *Lactobacillus acidophilus*, using three different antibacterial tests, namely: Disk agar diffusion test, Biofilm inhibition test and eluted component test. For evaluating the shear bond strength, 80 extracted premolars were collected and divided into four groups (n=20 each), which were bonded with stainless steel preadjusted Edgewise brackets, by using these modified composites. Their SBS was then compared with that of the control group, using a universal testing machine.

**Results:**

Composite discs containing nanoparticles and fluoride were capable of producing growth inhibition zones for all bacterial types. Results of the biofilm inhibition test showed that all the study groups inhibited the bacterial count, in comparison to the control group. A significant difference of SBS was observed between all groups.

**Conclusion:**

The antibacterial activity of orthodontic composites modified with Ag and TiO_2_ nanoparticles was significant, compared with conventional and fluoride-containing composites. The control group showed the highest SBS, followed by fluoride, titanium, and silver groups, with statistically significant difference in mean SBS values among all groups.

## RESUMO

**Objetivo:**

O objetivo do presente estudo foi determinar a eficácia das propriedades antibacterianas de compósitos contendo nanopartículas de prata (Ag NPs) ou nanopartículas de dióxido de titânio (TiO_2_ NPs) e de um compósito com liberação de flúor, contra as bactérias *Streptococcus mutans* e *Lactobacillus acidophilus* e, também, avaliar o efeito na resistência ao cisalhamento (RC) desses compósitos modificados com nanopartículas.

**Métodos:**

Os compósitos ortodônticos foram modificados pela adição de 1% p/p de Ag NPs e 1% p/p de TiO_2_ NPs. Discos de compósito foram preparados para avaliar as propriedades antibacterianas desses grupos contra as bactérias *Streptococcus mutans* e *Lactobacillus acidophilus*, usando três testes antibacterianos diferentes: teste de disco-difusão em ágar, teste de inibição da formação de biofilme e teste de componentes eluídos. Para avaliar a resistência ao cisalhamento dos compósitos, 80 pré-molares extraídos foram coletados e divididos em quatro grupos, contendo 20 dentes cada. Foram utilizados braquetes Edgewise pré-ajustados de aço inoxidável para colagem nos dentes usando as resinas modificadas. A RC desses compósitos modificados foi comparada à dos grupos controle, usando uma máquina universal de testes.

**Resultados:**

Os discos de compósito contendo as nanopartículas e flúor foram capazes de produzir halos de inibição de crescimento para todos os tipos de bactérias. Os resultados do teste de inibição do biofilme mostraram que houve uma redução na contagem de bactérias em relação ao grupo controle. Diferenças significativas na RC foram observadas entre todos os grupos.

**Conclusão:**

A atividade antibacteriana dos compósitos ortodônticos modificados com nanopartículas de Ag e TiO_2_ foi significativa, quando comparada ao compósito convencional e ao compósito com flúor. O grupo controle apresentou a maior RC, seguido pelos grupos Flúor, Titânio e Prata, com diferença estatisticamente significativa nos valores médios da RC entre todos os grupos.

## INTRODUCTION

Demineralization of the enamel surface during fixed orthodontic treatment was a common complication encountered in orthodontic patients.^1^ Brackets and fixed orthodontic attachments facilitate plaque accumulation by providing retentive areas.^2^ *Streptococcus mutans* and *Lactobacillus* causes a rapid shift in the microflora of plaque, resulting in elevated levels of acidogenic environment.^3^ The acidic byproducts of these bacteria in plaque are responsible for the subsequent enamel demineralization and formation of white spot lesions.^4^

The use of specific nanoparticles (NPs) as antibacterial agents has attracted much attention recently, in the fields of medicine and dentistry.^5^ The physicochemical nature of these NPs enables them to interact with the negatively charged surface of bacterial cells to a greater extent, resulting in enhanced antibacterial activity.^6^ *Streptococcus mutans* are known to be sensitive to nanoparticles of silver, zinc oxide, gold and titanium, and significant clinical effects can be observed with the use of these nanoparticles.^7^

Ag NPs have distinctive characteristics that make them a possible choice to be used as fillers for dental composites.^8^ TiO_2_ plays a vital role in organic degradation processes, due to its properties, such as biocompatibility and chemical stability.^9^ TiO_2_ NPs, when investigated, exhibited superior antibacterial activity against *Streptococcus mutans*.^10^

The concentration and distribution of nanofiller particles into the orthodontic adhesives are critical parameters that affect the antibacterial properties and their shear bond strength (SBS). Previous studies^11^ ^-^ ^13^ have shown that the addition of 1%, 5% or 10% w/w concentrations of silver/hydroxyapatite NPs to the orthodontic adhesives increased the antibacterial properties, but simultaneously affected the SBS. The incorporation of 1% and 5% w/w of these NPs maintained the SBS of orthodontic adhesives, whereas increasing its concentration up to 10% w/w significantly decreased the SBS of orthodontic adhesives.^11^ ^-^ ^13^

Thus, the present study aimed at incorporating 1% (w/w) TiO_2_ NPs and 1% (w/w) Ag NPs into a commonly used orthodontic adhesive, evaluating the antibacterial properties and the SBS of the modified composites, and comparing it with a fluoride-releasing composite and a conventional composite.

## MATERIAL AND METHODS

### Nanoparticles used for the study

» TiO_2_ NPs (dry nanopowder, anatase phase, average primary particle size: 30-50 nm; purity: > 99.5%, Nano Research Lab, Jamshedpur, Jharkhand, India).
» Ag NPs (dry nanopowder, average primary particle size: 30 - 50 nm; purity: > 99.5%, Nano Research Lab, Jamshedpur, Jharkhand, India).

### Adhesives used for the study

» Conventional orthodontic composite (Enlight, Ormco Corp, CA).
» Fluoride-releasing composite (Light Bond, Reliance Ortho Products, Illinois, USA).

### Media used for bacterial culture

» Mueller-Hinton agar (MHA) media (Hi-Media Laboratories Pvt. Ltd, Visakhapatnam, Andhra Pradesh, India).
» Brain-Heart Infusion broth (BHI broth) (Hi-Media Laboratories Pvt. Ltd, Visakhapatnam, Andhra Pradesh, India).

### Types of bacterial strains used

»*Streptococcus mutans* (MTCC 497), CSIR - Institute of Microbial Technology, Chandigarh, Punjab, India.
»*Lactobacillus acidophilus* (MTCC 10307), CSIR - Institute of Microbial Technology, Chandigarh, Punjab, India.

### Equipment used

» Composite mixer - High Energy Ball Mill (Retsch-Emax, Centre for Nanotechnology & Centre for Excellence, Andhra University, Visakhapatnam, Andhra Pradesh, India).
» Scanning Electron Microscope (JEOL JSM-881 OSV, Advanced Analytical Laboratory DST, Andhra University, Visakhapatnam, Andhra Pradesh, India).
» Universal testing machine: Instron (model-8801, Norwood, MA, USA).

### Methodology

This study consisted of 1 control group and 3 experimental groups:

» Group I: control group - conventional composite.
» Group II: titanium group - composite containing 1% w/w TiO_2_ NPs.
» Group III: silver group - composite containing 1% w/w Ag NPs.
» Group IV: fluoride group - fluoride-releasing composite.

## NANOCOMPOSITES PREPARATION

**Figure 1:**
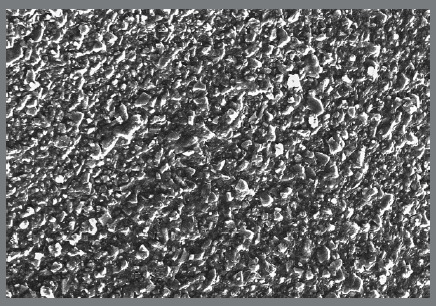
SEM image showing uniform distribution of TiO_2_ NPs, at 200x magnification

To achieve a concentration of 1% TiO_2_ (w/w) in the orthodontic adhesive, 40mg of TiO_2_ NPs were added to 4000 mg of orthodontic adhesive (Enlight, Ormco Corp, CA) and blended by using a composite mixer (High Energy Ball Mill, Retsch - Emax, Centre for Nanotechnology & Centre for Excellence, Andhra University, Visakhapatnam, Andhra Pradesh, India) at a speed of 3500 revolutions per minute in dark environment for 5min. A scanning electron microscopy (SEM) examination at a magnification of 200x was performed on a cured sample, to check the uniform distribution of the TiO_2_ NPs within the composite paste^14^ (Fig 1).

**Figure 2:**
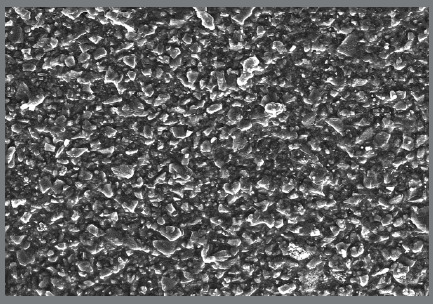
SEM image showing uniform distribution of Ag NPs, at 200x magnification.

For the preparation of 1% Ag (w/w) nanocomposite, the same above-mentioned procedure was followed, then a cured sample was examined on a scanning electron microscope at a magnification of 200x, to confirm the uniform distribution of Ag NPs within the composite paste (Fig 2).

### PREPARATION OF SAMPLES FOR TESTING ANTIBACTERIAL ACTIVITY

A total of 800 composite discs were used for the study. The thickness of these composite discs was set to 3mm, with diameter of 6mm, using a clear transparent thermoplastic sheet (Bioplast, Libral traders, New Delhi, India), and holes of specific dimensions were made using a high-speed air rotor handpiece.

Discs prepared with the four types of composites were used to evaluate the antibacterial activities against *Streptococcus mutans* (MTCC 497) and *Lactobacillus acidophilus* (MTCC 10307).

### EVALUATION OF ANTIBACTERIAL ACTIVITY

#### Disk agar diffusion test (DAD)

This test determines the ability of antibacterial agents to diffuse within agar and produce a bacterial inhibition zone. The composite discs (n=4) were placed on the Mueller-Hinton agar (MHA) plate, at a distance of 2 cm from each other, and then 20 µL from the bacterial suspensions (?10^8^ CFU/ml) were spread, whose density was adjusted with sterile phosphate buffer saline (PBS) to match its density to 0.5 in McFarland scale. 

**Figure 3:**
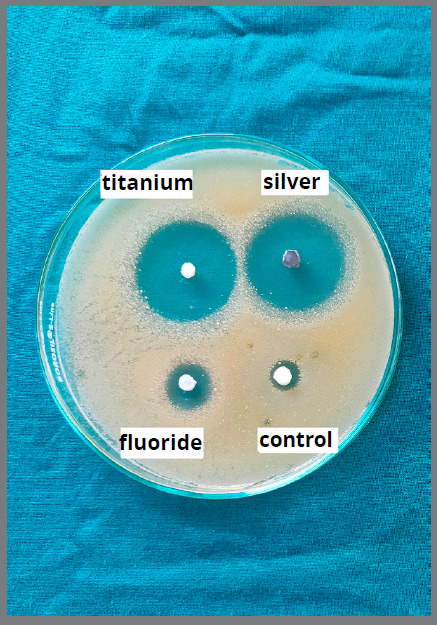
Bacterial growth inhibition zones for *S. mutans*, using DAD test.

**Figure 4:**
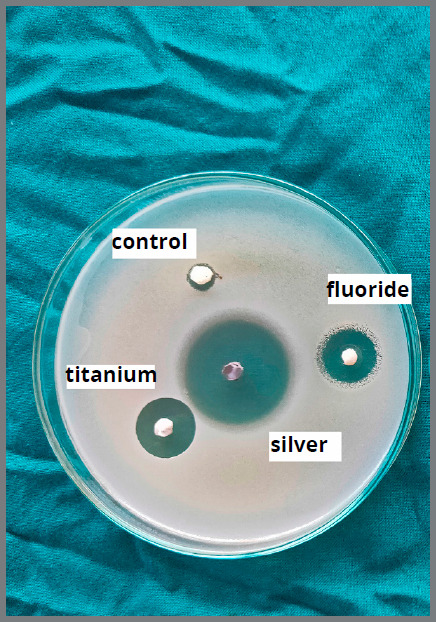
Bacterial growth inhibition zones for *L. acidophilus*, using DAD test.

After 48 hours of incubation, the bacterial growth inhibition diameter for both *S. mutans* and *L. acidophilus* was measured using an inhibition zone measuring scale (Figs 3 and 4).

### Biofilm inhibition test

Three-day biofilms were generated on composite discs (n=4) using flat-bottom 96-well microtiter plates (TCP177), which were later inoculated with the adjusted bacterial inoculum. Once the 10 µL of bacterial suspension was added, biofilms were incubated at a temperature of 37°C for 72 h. At the end of the third day, composite discs were removed and rinsed thoroughly with sterilized saline, to wash out the planktonic and loosely attached cells. Finally, in order to dislodge biofilms, all the composite discs were immersed in 1ml BHI broth and vortexed for 1 min. The CFUs/mm^2^ of test wells were calculated using the Miles et al.^15^ method.

### Antibacterial properties of eluted components

The discs were immersed in tubes containing 5 ml of BHI broth at 37°C. Discs were removed, and liquid media were transferred to new plastic tubes after 2 hours, 3 days and 30 days. The density of the bacterial suspension was adjusted to match 0.5 in the McFarland scale (≈10^8^ CFU/ml). Fifty microliters of bacterial suspension (in a final concentration of 2.5 × 10^5^ CFU/ml) were added to the previous tubes, and tubes were agitated at 300 rpm for 24 h at 37°C. The CFUs/ml of test wells were calculated using the Miles et al.^15^ method.

### EVALUATION OF SHEAR BOND STRENGTH

Eighty premolars freshly extracted for orthodontic treatment purposes were divided into four groups of 20 teeth each. The inclusion criteria for tooth selection was: anatomically and morphologically well-defined extracted premolars, with intact buccal enamel surface, and without any developmental defects, enamel caries, or fractured crowns. Then the samples were mounted in cold-cure acrylic resin poured in PVC tubes. The teeth were vertically embedded in acrylic up to the cemento-enamel junction (CEJ), and standard bonding procedure was carried out.

The buccal surface of the tooth was etched using 37% phosphoric acid (Eazetech, Anabond) for 30 seconds, rinsed thoroughly with running water for 30 seconds, and gently dried with air spray. A thin coat of primer (Orthosolo, Ormco, CA) was then applied with applicator tip and light-cured for 10 secs, followed by bonding of MBT 0.022-in stainless steel premolar brackets (Ortho Organizers) with the four types of orthodontic adhesives, light-cured for 40 seconds (10 seconds on each side). While bonding the brackets to the tooth surface, a 300-g force was applied for approximately 5 seconds, to ensure a uniform thickness of the adhesive, measured with a dynamometer (Dial tension gauge, MISUMI INDIA Pvt Ltd, Haryana, India). Excessive adhesive was removed with a probe. After completion of the bonding procedure, the teeth were immersed in a container with distilled water, for 24 hours.

Eighty samples from each of the four groups were subjected to a shear bond strength test, using an universal testing machine (Instron, model 8801). Testing was performed at a crosshead speed of 1mm/minute. The values obtained for the force required to shear the bracket, causing bond failure, were recorded in Mega Pascals (MPa).

## STATISTICAL ANALYSIS

Shear bond strength results were analyzed using Kruskal-Wallis test, as the standard deviation values were not less than half of the mean value, which means the data did not follow normal distribution. The results of the antibacterial tests have been analyzed using one-away ANOVA, followed by Tukey’s *post-hoc* test, used to find statistical significant differences between and within the groups. A *p*-value of less than 0.05 (*p*<0.05) was considered statistically significant, at a 95% confidence interval.

## RESULTS

### DISK AGAR DIFFUSION TEST

Statistically significant difference was seen in the mean antibacterial activity against both *S. mutans* and *L. acidophilus*. The mean diameter of bacterial inhibition zone showed that the silver group (Group III) presented the highest mean, and it was reduced for titanium group (Group II), followed by fluoride group (Group IV) and the control group (Group I) (Table 1).

**Table 1:** Comparison of disk agar diffusion test (mm) between the groups.

	Group	n	Mean	SD	***p*-value**
*S. mutans*	Group I	20	0.0000^#^	0.000	< 0.001
	Group II	20	6.850^#^	1.69	
	Group III	20	10.85^#^	2.94	
	Group IV	20	4.700^#^	2.51	
*L. acidophilus*	Group I	20	0.0000^#^	0.000	< 0.001
	Group II	20	7.3000^#^	1.78	
	Group III	20	10.700^#^	1.49	
	Group IV	20	4.150^#^	2.87	

One-way ANOVA , p<0.05; considered statistically significant.

# Pairs were significantly different from each other.

### BIOFILM INHIBITION TEST

Significant difference (*p*<0.05) was seen in mean bacterial colony count (CFU/mm^2^) in both *S. mutans* and *L. acidophilus*. The silver group (Group III) showed the lowest mean bacterial colony count, followed by the titanium group (Group II) ; and the mean bacterial colony count was increased in both the fluoride (Group IV) and control groups (Group I) (Table 2).

**Table 2:** Comparison of colony count (CFU/mm^2^) of *Streptococcus mutans* and *Lactobacillus acidophilus* between the groups, in biofilm inhibition test.

	Group	n	Mean	SD	***p*-value**
*S. mutans*	Group I	20	899.30*	134.12	< 0.001
	Group II	20	454.60*	176.51	
	Group III	20	315.70	127.39	
	Group IV	20	678.45	91.288	
*L. acidophilus*	Group I	20	870.00*	109.88	< 0.001
	Group II	20	440.85#	106.48	
	Group III	20	323.05#	189.30	
	Group IV	20	644.55*	177.95	

One-way ANOVA , p<0.05; considered statistically significant.

*, # Pairs were not significant in multiple pairwise comparison using Tukey’s test.

### ELUTED COMPONENT TEST

Significant difference was seen in the mean antibacterial activity for all groups, except on Day 0, when a significant reduction was observed in the colony count (CFU/ml) for all the groups from day 0 to day 3. A subsequent rise in colony count was seen between days 3 and 30, but comparatively the colony count on day 30 was less than that observed on day 0, except for the control group (Tables 3 and 4).

**Table 3:** Comparison of colony count (CFU/ml) of *S. mutans* between groups and time points in eluted component test.

Day	Group	n	Mean	SD	***p*-value**
0	Group I	20	2.310	0.126	0.25
	Group II	20	2.186	0.409	
	Group III	20	2.202	0.337	
	Group IV	20	2.225	0.207	
3	Group I	20	2.405	0.102	< 0.001
	Group II	20	1.908*	0.202	
	Group III	20	1.765	0.209	
	Group IV	20	2.060*	0.405	
30	Group I	20	2.680	0.128	< 0.001
	Group II	20	2.017*	0.207	
	Group III	20	1.922*	0.237	
	Group IV	20	2.105	0.391	

One-way ANOVA, p<0.05 considered statistically significant.

* Pairs were not significant in multiple pairwise comparison using Tukey’s test.

**Table 4:** Comparison of colony count (CFU/ml) of *L. acidophilus* between groups and time points in eluted component test.

Day	Group	n	Mean	SD	***p*-value**
0	Group I	20	2.1040	0.138	0.32
	Group II	20	1.9205	0.242	
	Group III	20	1.8155	0.288	
	Group IV	20	2.0880	0.231	
3	Group I	20	2.2095	0.135	< 0.001
	Group II	20	1.6110*	0.224	
	Group III	20	1.5630*^#^	0.243	
	Group IV	20	1.8305^#^	0.241	
30	Group I	20	2.4165	0.136	< 0.001
	Group II	20	1.7150*	0.221	
	Group III	20	1.6465^#^	0.256	
	Group IV	20	1.9320^#^*	0.268	

One-way ANOVA , p<0.05 considered statistically significant.

*, ^#^ Pairs were not significant in multiple pairwise comparison using Tukey’s test.

### SHEAR BOND STRENGTH

Statistically significant difference (p<0.005) was seen in mean shear bond strength values of all the groups. The mean SBS of the control group was the highest among the groups, followed by the fluoride, titanium, and silver groups (Table 5).

**Table 5:** Comparison of shear bond strength (MPa) between groups.

Group	n	Mean	SD	*p-* **value**
Group I	20	24.679#	6.400	0.005
Group II	20	17.177#	8.349	
Group III	20	14.694#	6.688	
Group IV	20	20.872#	9.869	

Kruskal-Wallis test, p<0.05; considered statistically significant.

^#^ Pairs are significant different from each other pair.

## DISCUSSION

Decades since its introduction, composite resin adhesives remain the first choice of most orthodontists for bonding brackets; however, they may facilitate demineralization of adjacent enamel. Some of their shortcomings have not been eliminated yet. De Soet and De Graaff ^16^ suggested that *Streptococcus mutans* is the main bacteria responsible for enamel demineralization.

Various anti-bacterial agents have been incorporated into the orthodontic adhesives to increase their antibacterial activity.^17^ ^,^ ^18^ But few studies^19^ ^,^ ^20^ have reported lower bond strength with the use of fluoride-releasing composite resins for bonding orthodontic attachments, for preventing demineralization.

With the advent of nanotechnology, and their excellent properties in orthodontic bonding, NPs have been widely used in biological and pharmaceutical applications. Kim et al.^21^ concluded that NPs and their ions can produce free radicals, resulting in the induction of oxidative stress (i.e., reactive oxygen species; ROS); the produced ROS can irreversibly damage bacteria (e.g. their membrane, DNA, and mitochondria), resulting in bacterial death.

Various authors have demonstrated that the incorporation of nanoparticles into the orthodontic adhesives affects their shear bond strength. However, an increase in its concentration has been considered cytotoxic.^22^ ^,^ ^23^

Nanoparticles are known for their antimicrobial properties because of their small size and increased surface area.^24^ They are also insoluble with a size smaller than 100nm.^9^ In this present study, a concentration of 1.0% w/w NPs was added to the orthodontic adhesives, as the small particle size and large surface area enables them to release more ions at a low filler level without being cytotoxic.

### Antibacterial activity

Disc agar diffusion test has shown significant growth inhibition zones against *S. mutans* and *L. acidophilus* with modified composites containing 1% w/w silver nanoparticles, 1% w/w TiO_2_ nanoparticles, when compared with fluoride-releasing and conventional composites. In the biofilm inhibition test, when the colony count (CFU/mm^2^) was calculated, the Ag group showed the highest antibacterial activity among all the four groups. Antibacterial properties of eluted components against *Streptococcus mutans* and *Lactobacillus acidophilus* at regular intervals of time showed a significant reduction in colony count (CFU/ml) of bacteria for the first three days in NPs-modified composites. Later a subsequent increase in colony count (CFU/ml) was observed from day 3 to day 30 in all the three experimental groups. 

This study demonstrated that 1% (w/w) Ag NPs have the highest antibacterial effect, which was in agreement with the results of the study conducted by Kassaee et al.^25^

Ag NPs have peculiar chemical, physical and biological properties, compared to those of traditional bulk materials. Their small particle size along with large surface area provide much more efficacious antibacterial properties.^12^ The reason behind reduced bacterial adhesion might be the reduced surface free energy (SFE).

The results of the present study demonstrated that 1% (w/w) TiO_2_ NPs significantly reduced bacterial growth, which is in agreement with the results of Sodagar et al.^26^

Fluoride-releasing composite showed statistically significant antibacterial activity, compared to the control group, and the results correlate with those of the study conducted by Swapna et al.^27^, which stated that the fluoride-releasing composite exhibit significant inhibition of demineralization, compared to the conventional bonding agents.

### Shear bond strength

In this study, the SBS of orthodontic adhesives modified with 1% (w/w) Ag NPs or 1% (w/w) TiO_2_ NPs, fluoride-releasing composite (Light Bond, Reliance Orthodontic Products) and the control group was compared.

It was observed that the mean SBS values of 1% (w/w) Ag NPs and 1% (w/w) TiO_2_ NPs groups were decreased, when compared with the fluoride-releasing composite and the control group. Though both silver and titanium nanoparticles were added in the same concentration in this study, the Ag group showed a significant decrease in SBS, compared to TiO_2_ group. Akhavan et al.^13^ evaluated that a decrease in bond strength associated with the addition of silver NPs could be due to the agglomeration of particles, creating defect points, and interfering with the curing process of the adhesive.

According to Reynolds^28^, the clinically acceptable range for SBS is 6 to 8 MPa. The present study showed that the mean SBS values of the experimental groups was greater than the clinically acceptable range, but smaller then the control group (18 to 20 MPa), which was in agreement with the study conducted by Reddy et al.^29^

Pseiner et al.^30^ reported that SBS of fluoride-releasing composite had provided sufficient mean bond strength, which was still less than the control group, and it may be used as an additional prophylactic measure in orthodontic therapy, which supports the results of the present study.

## CONCLUSION

Incorporating antibacterial agents like silver and titanium dioxide nanoparticles into the orthodontic adhesives has improved their antibacterial activity better than the commercially available fluoride-releasing and the conventional composites. However, there is a significant difference in the antibacterial activity and shear bond strength of nanocomposites. The Ag nanocomposite showed statistically significant better antibacterial activity than the TiO_2_ nanocomposite. 

There was a statistically significant difference in mean SBS values among the four groups. The control group showed the highest SBS, followed by fluoride, titanium, and silver groups.
